# A scoping review of methods to measure and evaluate citizen engagement in health research

**DOI:** 10.1186/s40900-022-00405-2

**Published:** 2022-12-10

**Authors:** Anmol Shahid, Inara N. Lalani, Brianna K. Rosgen, Bonnie G. Sept, Shelly Longmore, Jeanna Parsons Leigh, Henry T. Stelfox, Kirsten M. Fiest

**Affiliations:** 1 Department of Critical Care Medicine, Cumming School of Medicine, University of Calgary, Calgary, AB Canada; 2 Department of Community Health Science and O’Brien Institute for Public Health, Cumming School of Medicine, University of Calgary, Calgary, AB Canada; 3 Alberta Health Services, Calgary, AB Canada; 4 Dalhousie University, Halifax, NS Canada; 5 Department of Psychiatry and Hotchkiss Brain Institute, Cumming School of Medicine, University of Calgary, Calgary, AB Canada

**Keywords:** Citizen engagement, Patient and public involvement, Co-designed research, Community-based participatory research, Scoping review

## Abstract

**Background:**

Citizen engagement, or partnering with interested members of the public in health research, is becoming more common. While ongoing assessment of citizen engagement practices is considered important to its success, there is little clarity around aspects of citizen engagement that are important to assess (i.e., what to look for) and methods to assess (i.e., how to measure and/ or evaluate) citizen engagement in health research.

**Methods:**

In this scoping review, we included peer-reviewed literature that focused primarily on method(s) to measure and/or evaluate citizen engagement in health research. Independently and in duplicate, we completed title and abstract screening and full-text screening and extracted data including document characteristics, citizen engagement definitions and goals, and methods to measure or evaluate citizen engagement (including characteristics of these methods).

**Results:**

Our search yielded 16,762 records of which 33 records (31 peer-reviewed articles, one government report, one conference proceeding) met our inclusion criteria. Studies discussed engaging citizens (i.e., patients [n = 16], members of the public [n = 7], service users/consumers [n = 4], individuals from specific disease groups [n = 3]) in research processes. Reported methods of citizen engagement measurement and evaluation included frameworks, discussion-based methods (i.e., focus groups, interviews), survey-based methods (e.g., audits, questionnaires), and other methods (e.g., observation, prioritization tasks). Methods to measure and evaluate citizen engagement commonly focused on collecting perceptions of citizens and researchers on aspects of citizen engagement including empowerment, impact, respect, support, and value.

**Discussion and conclusion:**

We found that methods to measure and/or evaluate citizen engagement in health research vary widely but share some similarities in aspect of citizen engagement considered important to measure or evaluate. These aspects could be used to devise a more standardized, modifiable, and widely applicable framework for measuring and evaluating citizen engagement in research.

**Patient or public contribution:**

Two citizen team members were involved as equal partners in study design and interpretation of its findings.

***Systematic review registration*:**

Open Science Framework (10.17605/OSF.IO/HZCBR).

**Supplementary Information:**

The online version contains supplementary material available at 10.1186/s40900-022-00405-2.

## Plain English Summary

Involving members of the public (citizens) in health research is important. It helps make sure that research focuses on issues that are most important to citizens. It also helps ensure that the research done is respectful of citizen participation and most likely to provide benefit. However, the best way to engage citizens in research is unclear. In this scoping review, we examined existing studies that assessed citizen engagement in health research. We found that citizen engagement was often assessed by asking for feedback from both citizens and researchers. Feedback was collected in person (one on one interviews or group discussions) or in writing (using surveys or audits). Frameworks (organized ways of thinking about an issue) were also sometimes used to measure empowerment, impact, respect, support, and value of engaging citizens. It was clear from the frameworks that there is a need to develop clearer roles for citizens in research. The two citizen members of our research team who helped interpret our study findings felt that a set of guidelines for citizens to help them best participate in health research needs to be developed. We believe these observations could be used to create a more standard method for assessing citizen engagement in research.

**Supplementary Information:**

The online version contains supplementary material available at 10.1186/s40900-022-00405-2.

## Background

Citizen engagement in health research is an increasingly common approach to conducting biomedical, clinical, health system and services, social, cultural, environmental, and population health research with citizens as collaborators rather than subjects [1, 2]. Often referred to using diverse terminology (e.g., community based participatory research, public participation, patient and public involvement), citizen engagement recognizes citizens, defined as interested or affected members of the general public including patients, caregivers, advocates, or representatives of the community as “knowledge users”, or individuals who are affected by the processes and results of health research and can use their lived experience to influence research to be more relevant and useful [3, 4]. Specifically, citizen engagement encompasses meaningful involvement of citizens in various aspects of the health research process such as: membership in advisory groups or steering committees for priority-setting, co-application on funding grants, and research planning, decision-making, conduct, implementation, evaluation, and dissemination [3–5].

Engaging citizens in research has the potential to improve the relevance of study findings, minimize waste by facilitating stewardship over resources, create mutual learning and understanding, and build trust in research findings by improving relationships between communities and researchers [6, 7]. Additionally, citizen engagement has shown the ability to provide individuals with opportunities to acquire new skills and knowledge, enjoyment and satisfaction through support and friendship, and financial rewards to compensate their efforts [8]. Due to these documented benefits of citizen engagement, national funding bodies and healthcare organizations worldwide encourage and sometimes mandate citizen engagement in research design and practice [9, 10].

Despite the push towards incorporating citizen engagement in research by funding bodies, citizen engagement is often tokenistic and lacks the clarity and guidance needed to facilitate it’s meaningful use [11–19]. Existing guidance on citizen engagement is often provided within a “stakeholder engagement” context which is not specific to citizens and can include health care practitioners, policymakers and industry members and may not directly address the needs of citizens [20]. Finally, literature around citizen engagement tends to focus reiterating benefits, risks, and impact of citizen engagement in health research without detailing specifics on how to appraise citizen engagement in health research [1, 21–25]. As such, there is need for an evidentiary foundation to enable assessment of the degree (i.e., level of engagement in research processes which can vary from participation in research planning committees to recruitment of participants and dissemination of data) and quality (i.e., determining quality of involvement, which may be ascertained collecting citizens’ experiences with the engagement or perceived impact of engagement) of citizen engagement in health research, building upon current guidance provided by national funding agencies and peer-reviewed literature. To develop a high-level understanding of methodology used to appraise citizen engagement in health research and determine aspects of citizen engagement valuable to assess, we conducted a scoping review of literature focused on:

1. Methods to measure (i.e., determine degree of) citizen engagement in health research; and
2. Methods to evaluate (i.e., determine quality of) citizen engagement in health research.

## Methods

We designed and conducted a scoping review to map the existing literature on methods to measure and evaluate citizen engagement in health research according to the Arksey and O’Malley [26] and Levac [27] recommendations for scoping reviews. We used the Preferred Reporting Items for Systematic reviews and Meta-Analyses extension for Scoping Reviews (PRISMA-ScR) to guide the reporting of this scoping review [28]. A detailed description of the proposed methods has previously been published [29].

## Identification of the research question

As degree and quality of engagement are closely related features of citizen engagement, we developed the research question: “*what is the state of knowledge on methods to measure and evaluate citizen engagement in health research?*” to capture a broad range of potentially relevant literature. Our research question was developed to also shed light on the any relationship between measurement and evaluation of citizen engagement. We ensured that our research question defined the scope of inquiry with respect to population, concept, and outcomes of interest and would direct the subsequent steps [26].

We defined our target population as “citizens”, or consumers of health services (e.g., patients, families of patients, informal caregivers), advocates and representatives from community organizations, and members of the general public. Our target concept was “engagement”, “involvement”, or “participation” in health research. We used the CIHR definition of health research, which encompasses the biomedical, clinical, health systems and services, social health, cultural health, environmental health, and population health fields [30]. To complement our usage of the CIHR definition of health research with discussion around citizen engagement, we adopted the CIHR definition of citizen engagement or “the meaningful involvement of individual citizens…that is interactive and iterative with an aim to share decision-making power and responsibility for those decisions”. This definition encompassed activities such as priority-setting, planning, acquiring funding, research decision-making, research conduct (e.g., commenting on and developing research materials, interacting with research participants, and/or carrying out research activities), implementation, evaluation, or dissemination to be means of engagement [3].

### Identification of relevant studies

We identified relevant literature on citizen engagement in research using a pre-determined plan for data sources and search strategy, including search terms, languages, and dates of search. As per recommendations, we designed the search strategy to return reasonably relevant results while considering time and personnel workload as limiting factors [26, 27].

### Search strategy

Our study team, which included multiple stakeholders and knowledge users including health services researchers (KMF, HTS, JPL), trainees (AS, BKR), patient partners (BGS, SL), a health care professional (HTS), and a health sciences research librarian developed the search strategy. The search strategy (Additional file 1: Item S1) was independently reviewed by a second health sciences research librarian uninvolved with this project using the Peer Review of Electronic Search Strategies (PRESS) checklist [31].

Using the previously published search strategy [29], we searched the MEDLINE (Ovid) database from January 1, 2000 to February 1, 2021. We then adapted this strategy to each database to be searched: EMBASE (Elsevier) (January 1, 2000–February 1, 2021), Cochrane Library (Cochrane) (January 1, 2000–February 1, 2021), CINAHL Plus with full-text (Ebscohost) (January 1, 2000–February 1, 2021), APA PsycINFO (ProQuest) (January 1, 2000 – February 1, 2021), Scopus (Elsevier) (January 1, 2000–February 1, 2021), and Web of Science Core Collection (Clarivate) (January 1, 2000–February 1, 2021). We included subject headings, keywords and relevant synonyms related to three concepts: [1] citizens (e.g., community member, lay person, public, stakeholder), (2) engagement (e.g., collaboration, engagement, participation, involvement), and (3) health research (e.g., biomedical research, clinical research, public health research, environmental health research). We excluded studies published before the year 2000 to capture a modern viewpoint of the ever-evolving practice of citizen engagement in health research. We did not place any exclusion criteria on language. We screened the reference lists of included studies and related systematic reviews to identify additional potentially relevant literature.

### Study selection

We developed inclusion and exclusion criteria a priori through meetings with the study team to refine the study selection process at the beginning, midpoint, and endpoint of the citation screening process in case any unforeseen considerations arose [27]. We screened and selected relevant studies for inclusion in the scoping review independently and in duplicate.

### Eligibility criteria

We included articles if they: (1) were primary (e.g., observational or interventional studies) or secondary (e.g., systematic or scoping reviews) research, frameworks, reviews, or reports, (2) primarily focused on citizen engagement in health research (including biomedical, clinical, health systems and services, and social, cultural, environmental and population health, as defined by CIHR) and (3) reported method(s) to measure or evaluate citizen engagement. We did not place any restrictions on language. Non-English language studies were screened using Google Translate [32]. We excluded any literature that discussed methods to measure or evaluate citizen engagement in non-research processes, including health promotion, health education, health system or service delivery and governance (including decision-making), or health program implementation. To gain a focused insight on existing methods determining the degree and quality of citizen engagement in health research, we omitted literature focusing primarily on areas other than measurement and evaluation of citizen engagement in health research.

We imported retrieved articles into Covidence (Veritas Health Innovation, Melbourne, Australia) for title & abstract screening, which was completed independently and in duplicate by two reviewers (AS, BKR, KP, KK, RK, MA, ML, LH, KM). Reviewers conducted a pilot screening of 50 titles and abstracts to ensure consistency in application of inclusion and exclusion criteria. Once a Kappa (inter-rater agreement) of ≥ 0.8 was achieved, reviewers proceeded to screen the remaining articles. If one reviewer indicated an article as potentially relevant at the title & abstract screening phase, the article advanced to full-text review to ensure inclusivity. Following title & abstract screening, we exported a list of included articles into Endnote X9 (Clarivate Analytics, London, United Kingdom). We retrieved full-text versions of included articles using a combination of Endnote X9’s ‘find full text’ feature, Endnote Click online, and the local university online libraries. If a full-text version of an article was not available, a search was conducted of the publishing journal’s website to gain access. Following the search for full-text articles, the Endnote library was re-uploaded to Covidence and full-text screening was completed independently and in duplicate by two reviewers. Reviewers (AS, IL, RK) pilot screened the full text of 20 articles, and screened the remaining articles once a Kappa ≥ 0.8 was achieved. Both reviewers agreed on inclusion status and reason for exclusion at this stage. Any disagreements were resolved by discussion among the reviewers or the involvement of a third reviewer (IL or RK), if required.

### Charting the data

We developed an initial data charting form for data elements to be abstracted from the articles, then refined the form through discussion with the study team. The data charting form [Microsoft Excel (version 16.29.1)] was piloted by two reviewers (AS, IL) with ten included studies, and revised as needed. Once the final data charting form was developed, all relevant articles were abstracted independently and in duplicate by two reviewers (AS, IL). Abstracted variables in the initial data charting form included study characteristics, participants, type and goals of citizen engagement, method(s) used to measure citizen engagement, method(s) used to evaluate citizen engagement, features of method(s) used, and observed benefits or risks of the measurement or evaluation method(s) used.

### Collating, summarizing, and reporting the results

We collated, summarized, and reported the results by (1) analyzing the data, (2) reporting results, (3) and evaluating their meaning and aimed to contextualize our findings within existing literature and research, practice, and policy [26, 27]. We analyzed qualitative data specifically by having two research team members trained in qualitative methods (AS, IL) inductively code major components of each framework using an analytical approach informed by thematic analysis [33]. First, the researchers independently reviewed each framework to develop a list of relevant terms and associated concepts. Then, the researchers compared lists and discussed discrepancies with a third qualitative expert (JPL). The initial two researchers then deductively analyzed the agreed upon concepts into shared and unique framework features by expanding and collapsing shared meanings through a series of three meetings. Concepts deemed too vague (no label/definition provided) were excluded from analysis.

We present a descriptive summary of characteristics of the included documents, and the characteristics of the intended participants or audiences for these documents alongside a narrative synthesis of abstracted data variables.

### Consultation

Involvement of citizens in literature synthesis is recommended by funding organizations such as the NIHR [34] and CIHR [3] and is becoming increasingly commonplace [35, 36]. We involved citizens (BS, NF) in study conception and design including the search strategy. As per recommendations, we involved citizens (BS, SL) in the interpretation and contextualization of the data [27].

## Results

**Fig. 1:**
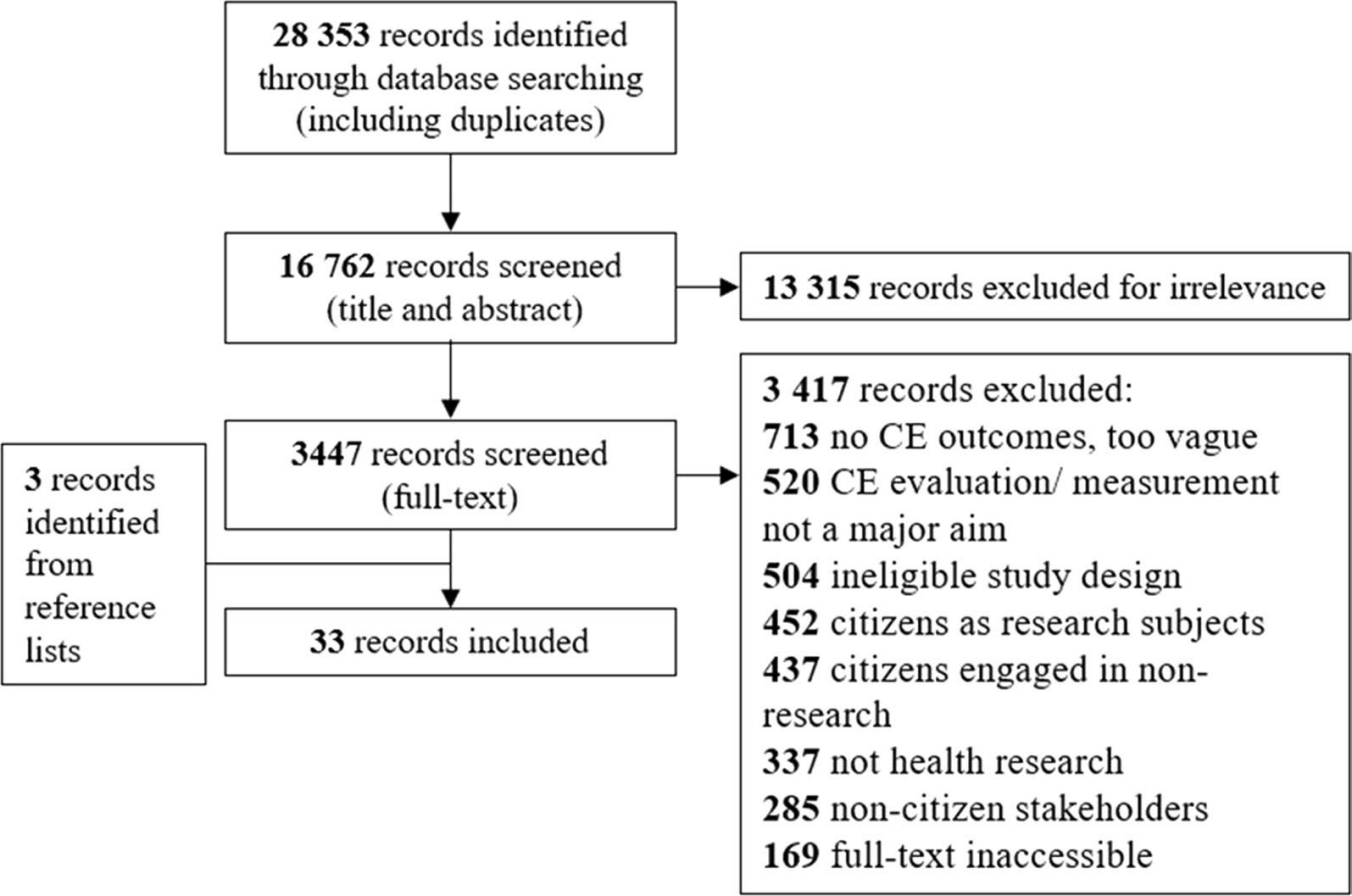
Flow diagram of included studies and reasons for exclusion

Our search strategy returned 28,353 total results (16,762 results after duplicates were removed). Of these, studies were excluded because they: (1) reported aspects of citizen engagement other than methods of measurement or evaluation (n = 713), (2) reported outcomes or discussion on specific diseases/interventions (i.e., not citizen engagement) (n = 520), (3) had an ineligible study design (i.e., editorials, letters, commentaries) (n = 504), (4) included citizens as health research subjects only (n = 452), (5) engaged citizens, but in non-research (i.e., health service design or delivery) (n = 437), (6) were not health research related (n = 337), (7) focused on non-citizen stakeholders in health research (i.e., clinicians, policy makers) (n = 285), or (8) and/ or had inaccessible full-texts (n = 169). Thirty records met inclusion criteria and were included in our scoping review. Three additional records were found through searching reference lists and included for a total of 33 records. A study flow diagram including reasons for exclusion is shown in Fig. 1.

### Study characteristics

A majority of the included literature was published within the last 10 years (2011–2021, n = 26, 78.8%). Literature included peer-reviewed journal articles (n = 31, 94.0%), a report by a government organization (n = 1, 3.0%), and a conferencing proceeding (n = 1, 3.0%). Most literature reported work that was conducted in the United Kingdom (n = 16, 48.5%), Canada (n = 6, 18.2%), and other European nations (i.e., Ireland, Germany, Sweden) (n = 6, 18.2%). Most included literature pertained to health services or health research (n = 18, 54.5%), and clinical research (particularly primary care and mental health) (n = 12, 36.4%). Characteristics of the included literature are shown in Table 1.

**Table 1:** Characteristics of the included literature (n = 33)

References	Country	Research area	Citizen engagement terminology	Citizen engagement activities	Types of citizens engaged	Stated goal
Abelson et al. [69]	Canada	Health services research	Public and patient engagement	General engagement	Public and patients	Evaluation
Abma et al. [41]	Netherlands	Health research	Patient involvement	Priority-setting	Patients	Evaluation
Archana et al. [54]	Nepal	Cardiovascular disease research	Stakeholder engagement	Inform	Patients and caregivers	Evaluation
Payne et al. [57]	Australia	Health services research	Consumer and community participation	Participation, consultation	Consumers, community representatives	Evaluation
Arora et al. [66]	USA	Health research	Community based participatory research	General engagement	Community partners	Measurement
Boivin et al. [64]	Canada	Health research	Patient and public engagement	General engagement	Patients, public	Evaluation
Boote et al. [44]	UK	Health research	Public Involvement	Grant development	Patients, patient representatives	Both
*Brady et al. [38]	UK	Health research	Public Involvement	Advisory board	Young people (children)	Evaluation
Brutt et al. [45]	Germany	Health research	Patient involvement	Study design	Patients	Both
Costello et al. [39]	Ireland	Pediatric Rheumatology	Patient and public involvement	Advisory board	Young patients (aged 10–20 years)	Evaluation
Crossing et al. [63]	Australia	Oncology	Consumer involvement	Research decision-making	People affected by cancer	Evaluation
Gibson et al. [58]	UK	Health services research	Patient and public involvement	Consultation	People with mental illness experience, disabled children, public	Evaluation
Giebel et al. [53]	UK	Health services research	Patient and public involvement	Dissemination, co-production	Older citizens, mental health patients	Evaluation
Greenhalgh et al. [42]	UK	Health research	Public involvement	Priority-setting, proposal development, study design and conduct, reporting, dissemination	Patients, public	Evaluation
Greer et al. [60]	Canada	Drug use	Community-based participatory research	Co-research, data collection	Peer leaders	Both
Hanley et al. [51]	UK	Health research	Consumer involvement	Priority-setting, study conduct, dissemination	Citizens	Measurement
Howe et al. [43]	UK	Primary care	Public Involvement	Proposal creation, study materials development, steering group	Volunteer citizens	Evaluation
Jewell et al. [61]	UK	Mental health	Patient and public involvement	Advisory board	People with mental illness experience	Evaluation
Johnson et al. [46]	UK	Palliative care	Public Involvement	Co-production, protocol development and study design, ethics application, interpretation, dissemination	Institute public members	Evaluation
Joosten et al. [68]	USA	Translational medicine	Community engagement	General engagement	Members of the public	Both
Lindenmeyer et al. [40]	UK	Diabetes, health services research	Consumer involvement	Priority-setting, decision-making, study conduct, analysis, dissemination, advisory/steering groups	People living with diabetes	Evaluation
Maccarthy et al. [59]	Ireland	Basic and preclinical health research	Patient and public involvement	Consultation, evaluation	People living with rheumatic disease	Both
Meyrick et al. [62]	UK	Sexual health	Patient and public involvement	General engagement	Unspecified	Measurement
Morrow et al. [67]	UK	Health research	Service user involvement	General engagement	Service users	Measurement
Oliver et al. [55]	UK	Health services research	Public Involvement	Collaboration, consultation	Unspecified	Both
Pelletier et al. [65]	Canada	Health research—physical activity	Patient and public engagement	Priority-setting	Community partners	Evaluation
Seeralan et al. [49]	Germany	Primary care	Patient and public involvement	Study materials development	Patients with depression history	Evaluation
†Shikako-Thomas et al. [52]	Canada	Pediatric neurology	Patient engagement	Priority-setting, data collection and analysis, interpretation, dissemination	CHILD-BRIGHT stakeholders	Evaluation
Stocks et al. [47]	UK	Primary care	Patient and public involvement	Study administration, document review, design own projects	Public group interested in research	Evaluation
Vat et al. [50]	Canada	Health research	Patient engagement	Study materials development, evaluation, data interpretation, dissemination, co-application	Patients	Measurement
Warner et al. [37]	Sweden	Mental health	Patient and public involvement	Protocol development	Refugee advisors	Evaluation
Wright et al. [56]	UK	Health research	User involvement	Consultation, collaboration, user-control	Service users	Both
Wyatt [48]	UK	Primary care, health services	Consumer involvement	Co-applicants, study conduct, study design	Service-users, carers	Both

*Report commissioned by a government organization

^†^Conference proceeding

### Citizen engagement: participants, engagement activities and terminology

**Fig. 2:**
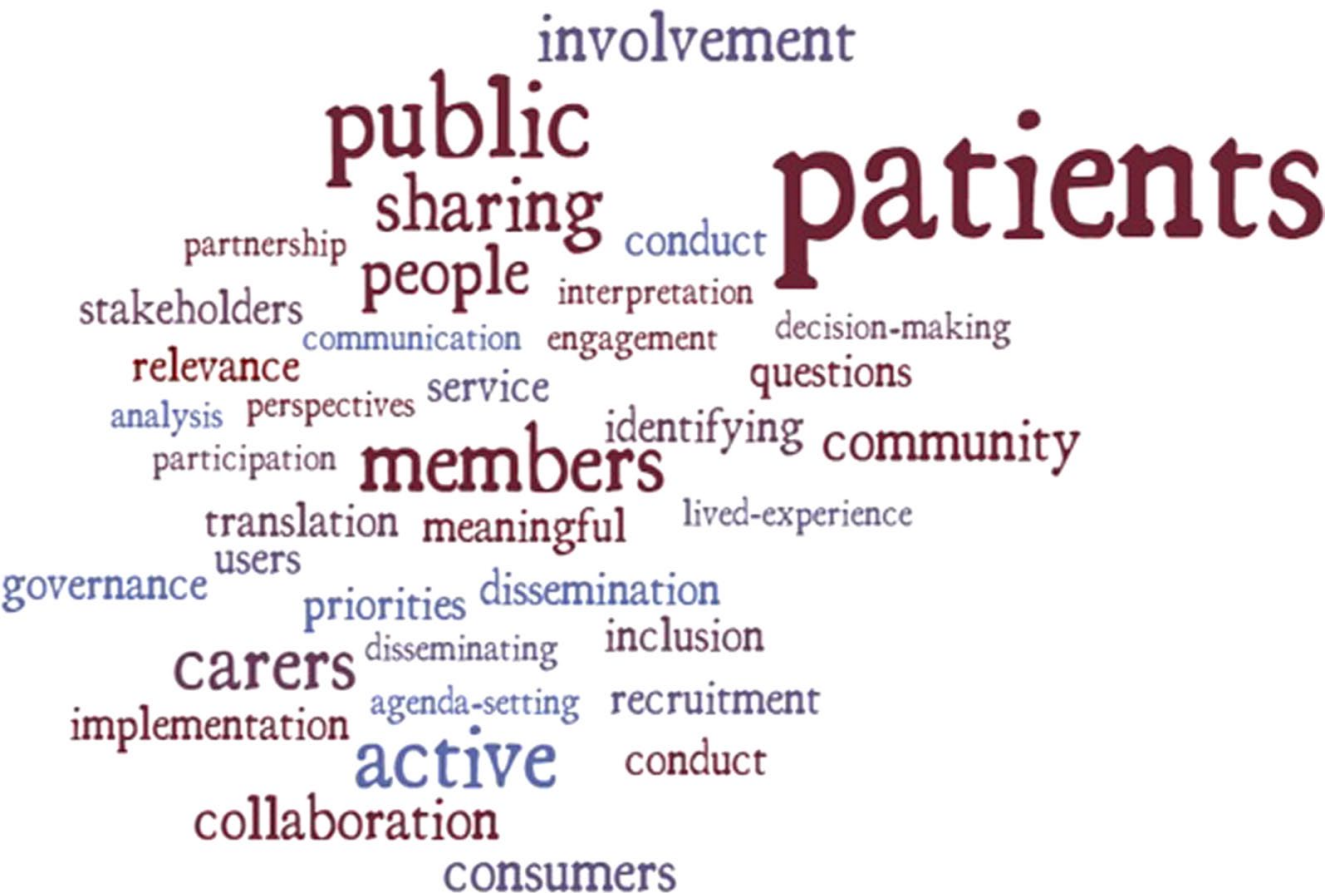
Word cloud of citizen engagement descriptions, activities, and key terms described in included literature

Included records varied in type of citizens engaged, research engagement activities, and terminology used to describe the engagement process (Table 1). Most studies reported engaging patients or individuals with lived experience of a given illness (n = 16, 48.5%), members of the public (n = 7, 21.2%), service users/consumers (n = 4, 12.1%), or individuals from community groups such as refugees [37] or children [38, 39] (n = 3, 9.1%). Citizens were engaged in specific research activities such as priority-setting [40–42], advisory board/ steering committee membership [38–40, 43], grant [44] and research proposal development [42, 43], study design [42, 45–48], study materials development [43, 49, 50], study administration and conduct [40, 47, 48, 51], interpretation of findings [46, 50, 52], and dissemination [40, 42, 46, 50–53]. Other studies described citizen engagement activities more generally as informing citizens of research goals so engagement may occur [54], collaboration [55, 56], consultation [55–59], co-production [46, 53], co-research [60], participation [57], and user control [56]. A word-cloud depicting descriptions of citizen engagement in the included studies is shown in Fig. 2.

Terminology used to describe citizen engagement activities (in order of most common to least common) was: patient and public involvement [37, 39, 47, 49, 53, 58, 59, 61, 62], public involvement [38, 42–44, 46, 55], consumer involvement [40, 48, 51, 63], patient and public engagement [64, 65], patient involvement [41, 45], and community-based participatory research [60, 66]. Table 1 shows specific studies, citizen engagement terminology, engagement activities, and types of citizens engaged in further detail.

### Methods used to measure and/or evaluate CE

Of the 33 included studies, 20 (61.0%) presented method(s) to evaluate citizen engagement, five (15.2%) presented method(s) to measure citizen engagement, and eight (24.2%) presented method(s) to both measure and evaluate citizen engagement. Methods for the measurement and/or evaluation of citizen engagement included frameworks, discussion-based methods (i.e., focus groups, interviews, workshops), survey-based methods (i.e., audits, questionnaires), and other methods (i.e., indicators, observation, prioritization tasks). Many studies utilized and reported on more than one method to measure and/or evaluate citizen engagement. A summary of these methods is presented in Table 2 and described narratively below.

**Table 2:** Methods used by included literature to measure and/ or evaluate citizen engagement

Method	Features	Content/theme(s)	References
*Framework*			
–	Four dimensional, theoretical	Weak voice/strong voice; One way to be involved/many ways to be involved Organization’s concerns/public’s concerns; Organization changes/organization resists change	[58]
Peer Engagement Process Evaluation Framework	Four process goals	Supportive environment, equitable participation, capacity building and empowerment, involvement in protocol developments, analysis, and outputs	[60]
Quality involvement Framework	Six dimensions	Ability to (i.e., access research resources, contribute, express views); Potential to (i.e., take up roles, identify and organize interests); Sense of being (i.e., valued as a partner not controlled, empowered); Research relationships (i.e., expectations); Ways of doing research (i.e., roles available to be taken up), Research structures (i.e., research organizations, ethics, governance)	[67]
Evaluation framework	Based on A Model Framework for Consumer and Community Participation in Health and Medical Research [79]	Impact and value of engagement, compliance with Telford’s principles	[57]
–	Conceptual framework	Degree of researcher versus public engagement	[55]
*Method (discussion based)*			
Focus group	Semi-structured	Experiences, impact, perceived purpose of patient and public involvement, recommendations	[46]
		Enjoyment/satisfaction from participation, perceived impact, impact of involvement on study	[45]
		Patients' need for information and layout needs	[49]
		Experiences, activities, perception of involvement (positive or negative), recommendations for involving others	[53]
		Issues of the restricted socio-demographics of the group, the perceived lack of feedback from researchers, and the effectiveness of current and future means to maximize volunteer support	[43]
		Perspectives on the project	[48]
	Semi-structured; with presentation of results	Reflection on practical guidelines, based on the preliminary results	[41]
Interview	Semi-structured	Views on monitoring and evaluation systems used to record involvement activities, feasibility of systematically collecting/collating data on the nature and impact of young people’s involvement, key opportunities and challenges	[38]
		Researchers views on impact of community expert input on their attitudes and practices	[68]
		Experiences with initiating and formulating the research agenda, verification of data obtained by study, experiences with programming and implementation, barriers and facilitators for patient involvement in programming and implementation	[41]
		Need for public involvement as a means to funding and recruitment, value of efficiency and the speed of turnaround of queries	[43]
		Perspectives of the project	[48]
Workshop	Included introduction of framework	Experiences of involvement as members of a public group within their parent organizations; mapping these along framework	[58]
*Method (question based) i.e., audit, questionnaire, survey)*			
CBPR Tool	Information not available	Satisfaction with involvement, trust, comfortability in sharing opinions	[52]
PPI audit tool	12 Questions; open-ended	Identifying PPI work (e.g., what is the PPI for? What is your current activity to involve users?) Effectiveness of PPI work (e.g., What is the goal of your PPI? Who is involved (how diverse) in PPI work at what) Resourcing PPI work (e.g., Who looks after PPI in your organization? What is their role?)	[62]
–	7 Questions; open-ended†	Motivation for involvement, feedback meeting structure and length, feedback on group, training required, project feedback, impact of involvement, value of involvement, general comments	[61]
–	5–6 Questions; open-ended†	Impact of public involvement, nature of involvement, information level, satisfaction	[44]
Partnership Assessment In community-based Research (PAIR)	31 Close-ended items (Likert scale) and one open ended item	communication, collaboration, partnership values, benefits, and evaluation, with an open-ended final item included to assess respondents’ view of whether they believe completion of the measure will impact how they work with their partner in the future	[66]
Public and Patient Engagement Evaluation Tool (PPEET)	Closed and open-ended questions†	Principles of ‘quality engagement’: (i) integrity of design and process; (ii) influence and impact; (iii) participatory culture; and (iv) collaboration and common purpose. Three unique questionnaires were developed to assess each of these four evaluation domains from the following perspectives: (i) those who participate in PPE activities; (ii) those who plan, execute or sponsor PPE activities within organizations; and (iii) those who provide the leadership and capacity for PPE within their organizations	[69]
Quality involvement questionnaire	4–7 Statements in each part; agreement rated on not at all to high	Multi-part questionnaire on perceived ability, potential, sense of being, research contexts, ways of doing research, research structure	[47, 67]
PPI assessment survey (Open-source evaluation tool)	19 Questions with 1–10 scale an open-ended option; 30 questions (not true at all to moderately true)	Satisfaction on (1) facilities, (2) level of contribution, (3) comfort, (4) understanding, (5) support and personal factors	[59]
–	12 Observable behaviours, paper-based assessment form using Likert scale	Interpersonal relations, nature of advisor contributions, how advisors guided research development	[37]
–	Structured; closed- and open- ended questions	Nature of this involvement in general terms, impact of that involvement, details of specific trials in which consumers had been involved, background of the consumers who had been involved, and the researcher's views about impact of consumer involvement	[51]
–	7 With statements based [1–10 scale], yes/no questions, one open-ended question †	Perceived value of consumer contribution, perceptions of consumer contribution, information about consumer contribution in previous projects (Researcher’s survey) Perceived value of contribution, perceived respect, empowerment, information, previous experience with contribution (Consumer’s survey)	[63]
–	13–16 Items; Forced or multiple choice (Likert scale), open-ended questions †	What changes they made to their research proposal, research project, or community engagement practices as a result of the input they received from the community experts, community expert experiences, process and outcomes, suggestions to improve the engagement process	[68]
Adapted from Patients as partners in research survey	Yes/no questions, close-ended questions, qualitative items †	Engagement evaluation, views on understanding, sensitivity, feasibility of project, appropriateness of project, satisfaction	[50]
Post-meeting survey	9 Questions (Likert scale); 4 open-ended questions	Evaluation of workshop, satisfaction, level of understanding, empowerment, support provided	[65]
–	Information not provided	Satisfaction, reasons for engagement (i.e., interest in research, desire to improve research to benefit patients, desire to use own experiences in a valuable way, personal learning), nature of involvement (i.e., advice on lay summaries, training activities for volunteers, membership of specific activity/ research group, support received from the staff of the project)	[43]
–	Open-ended questions, attitudinal statements (Likert scale) *	Motivation for involvement in research, role in research, reflections on consumer involvement	[48]
–	8–15 questions (5-point Likert scale) †	Content (format and structure, scope and meaning, characteristics of lecturers) Communication and supports for participation, sharing views and perspectives, impacts and influence, final thoughts, integrity and design of process, final reflections	[49]
Critical appraisal guidelines	9 Multi-part questions; open-ended	Rationale for involving users, recruitment strategy, training, methodology, value of citizen involvement, evaluation of impact	[56]
*Method (other)*			
Canadian Centre for Excellence on Partnerships with Patients and Public (CEPPP) evaluation tool	Tool to evaluate frameworks	Types of frameworks found (categorized) power‐focused; priority‐setting; study‐focused; report‐focused; and partnership‐focused	[42]
Observation		Observation (attending individual project steering group meetings)	[48]
PPI-ready planning canvas		Barriers, concerns, worries, challenges, value and impact, challenges in the implementation and adoption of PPI into research practice	[39]
Prioritization tasks		Prioritization of needs for feedback; evaluation of drafts	[49]
Telford’s Indicators of user involvement	16 Indicators, 8 principles	Roles of consumers agreed upon by researchers and consumers, budgeting appropriately for cost of consumer involvement, respecting consumers, support and necessary skills, consumers involved in decision making and dissemination, CE is reported on, research findings are made available and easy to understand)	[40]

^*^More than one reference to a study is presented in the event more than one method for the measurement or evaluation of citizen engagement was presented

^†^Indicates multiple intended audiences (i.e., intended for citizens, patients, researchers) or multiple versions of questionnaire/survey

### Citizen engagement strategies: frameworks

**Fig. 3:**
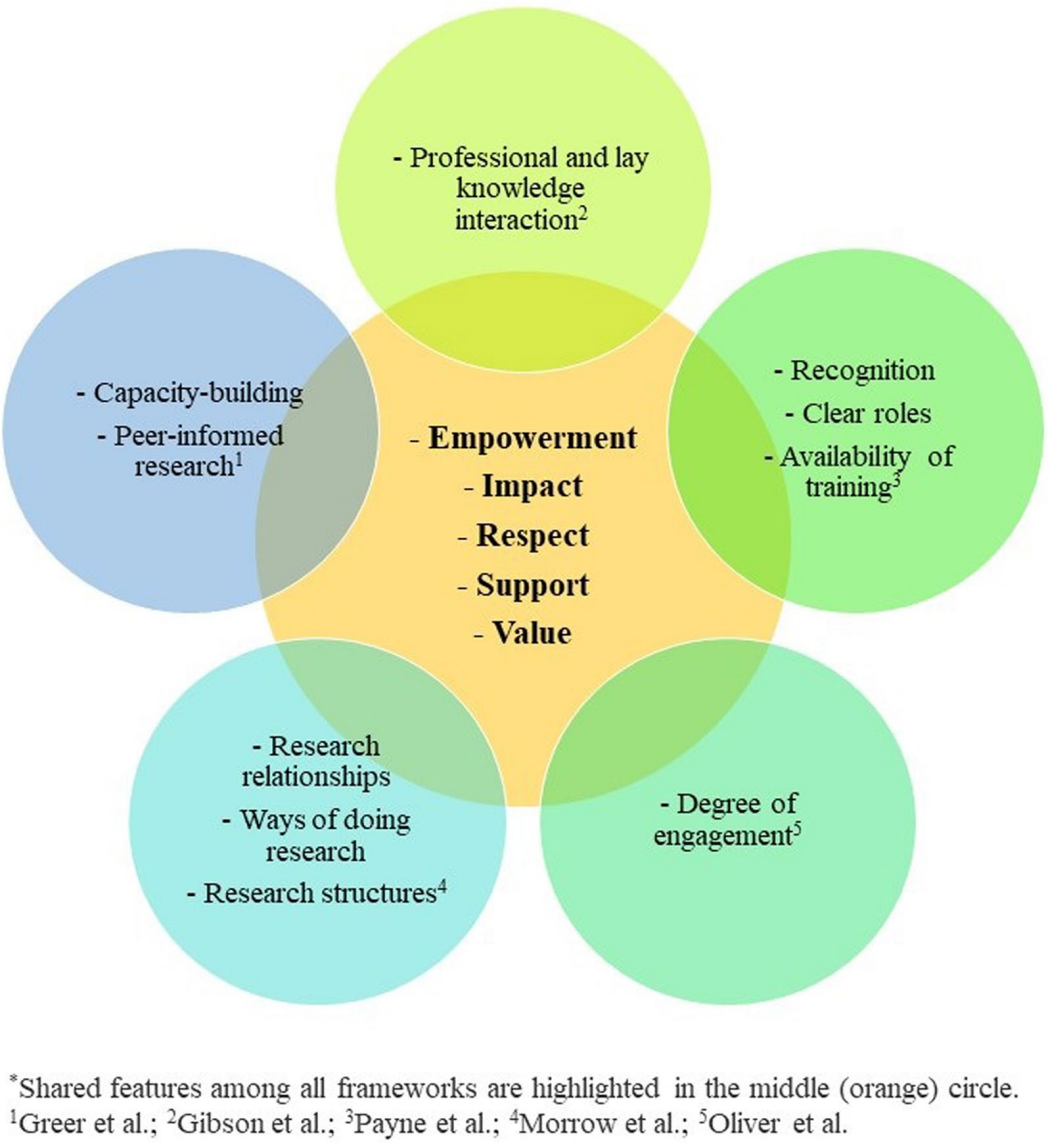
Similar and unique features of the five frameworks for measuring and/or evaluating citizen engagement included in this scoping review

Five studies presented frameworks [55, 57, 58, 60, 67] designed to measure and/or evaluate citizen engagement in health research. Frameworks focused on various aspects of citizen engagement including reflection on and impact of citizen engagement activities in research, and recommendations for improvement. The five included frameworks explored measurement and evaluation of citizen engagement through gauging (1) empowerment (i.e., citizens should feel comfortable in voicing their opinions), (2) impact (i.e., research should be positively shaped by citizen engagement), (3) respect (i.e., citizens should feel respected), (4) support (i.e., citizens should have training and supports available), and (5) value (i.e., citizens should feel important to the process). Included frameworks also highlighted the importance of capacity building (i.e., funds, personnel to support engagement in research) [60], assessing the degree of engagement of researchers and citizens [55], clarity in roles (i.e., of citizens when engaged) [57], and involvement of citizens in critical aspects of research (i.e., protocol development, analysis, outputs) [60]. More detail on each framework is provided in Table 2, and similarities and differences between the included frameworks are highlighted in Fig. 3.

### Citizen engagement strategies: discussion-based methods

Methods using discussion to measure and/or evaluate citizen engagement utilized focus groups (n = 7) [41, 43, 45, 46, 48, 49, 53], interviews (n = 5) [38, 41, 43, 48, 68], or workshops (n = 1) [58]. Most discussion-based measurement or evaluation of citizen engagement was conducted in a semi-structured manner, with pre-defined topics or question lists [38, 41, 43, 45, 46, 48, 49, 53, 68]. Common focus group discussions included experiences with and perceptions of involvement [46, 48, 53], nature and impact of involvement [45, 46, 53], and recommendations for improving involvement in research [43, 46, 53]. Common topics of discussion in interviews were experiences with involvement [38, 41], perspectives on the project in which involvement occurred [48], challenges in involvement [38, 41], and opportunities for improvement [38].

### Citizen engagement strategies: survey-based methods

A number of studies used survey-based methods to measure and/or evaluate citizen engagement in health research. These methods included an audit [62], questionnaires [37, 51, 59, 66, 67, 69], and surveys [48, 50, 51, 59, 63, 65] and varied in number and type of questions asked, content of the questions, and intended recipients of the questions. Open-ended questions [44, 48, 51, 56, 61–63, 65, 66, 68, 69] and Likert scale-based statements [37, 48, 49, 65, 66, 68] were commonly used. Many studies used a combination of open-ended questions, closed-ended questions, and/or statements for which a degree of agreement could be declared [48, 50, 51, 59, 63, 65, 66, 68, 69]. Questions were intended for (1) citizens involved in research [43, 44, 48, 50, 52, 61, 63, 65, 67, 69], (2) researchers who involved citizens in their work [44, 51, 52, 56, 59, 62, 63, 68, 69], or less frequently (3) other research or grant administrative personnel [52, 69]. The content of many questions focused on reflections on involvement (i.e., feedback on activity or study involved in) [44, 48–50, 52, 59, 61, 65], motivation for involvement in research [43, 48, 56, 61], perceived impact of involvement on the research [44, 49, 51, 61, 69], and recommendations or comments on future involvement in research [43, 63, 68].

### Citizen engagement strategies: other methods

A number of studies presented other methods to measure and/or evaluate citizen engagement. These included indicators of user involvement such as documentation of citizen roles in research and availability of training to citizens to facilitate their involvement in research [40], prioritization tasks focusing on outcomes of the research considered important by participating citizens [49], and citizen observation of any study steering group meetings and scrutiny of any study documentation [48]. One study used a method to appraise existing frameworks for supporting citizen engagement (the Canadian Centre for Excellence on Partnerships with Patients and Public evaluation tool) [42], however many of the frameworks discussed intended to *support and report* rather than measure or evaluate citizen engagement, falling out of the scope of this review.

## Discussion

Our scoping review produced two main findings. First, we found that multiple methods (i.e., audits, focus groups, interviews, frameworks, surveys) have been used, often in combination, to measure and evaluate citizen engagement in health research. These methods collect perceptions of citizens, researchers, and/or research support personnel on many aspects of citizen engagement including reasons, type, and impact of engagement, any challenges encountered in engagement (including project-specific issues), and recommendations for improving future citizen engagement in health research. Secondly, we identified that existing frameworks to measure and evaluate citizen engagement commonly assess perceived empowerment, impact, respect, support, and value. Together, these findings summarize the nature of citizen engagement in health research and itemize citizen engagement aspects that are considered important to assess the degree and quality of citizen engagement in health research.

In addition to our main findings, we identified that the terminology used to define citizen engagement and describe its activities varies widely. Citizen engagement is referred to as patient and public involvement (often in the United Kingdom), patient engagement, public engagement, consumer or service user-involved research, and community based participatory research depending on location and context. Varying terminology may pose a challenge for individual researchers to identify and utilize methods to appraise citizen engagement in research. Standardization of terminology could add to the accessibility and applicability of current and future methods to incorporate and evaluate citizen engagement in health research.

In the process of screening literature for inclusion in this scoping review, we found that much of the current guidance on appraising citizen engagement in research exists in the form of editorials, letters to the editor, commentaries, and perspectives from experienced researchers in the field. We noted that this type of literature does not routinely discuss the merit of discussion-based methods that were used to evaluate citizen engagement in included studies. This could reflect a repeated dismissal of discussion-based qualitative research methods to measure and evaluate citizen engagement in research and warrants further investigation. Despite limited discussion-based methods to appraise citizen engagement, this literature emphasizes the context and process of engagement [70], clarity, reflexivity, methodological rigour, transparency, pragmatism, and reciprocity as key principles to evaluating citizen engagement in research [71] and highlights the need for evaluation as an ongoing part of the research process [72]. These elements of citizen engagement complement our main findings and should be taken into consideration when appraising citizen engagement. Our findings also align with previous work emphasizing the importance of evaluating citizen engagement activities as a necessary step in building a strong evidence base for utilizing citizen engagement in health research [73]. Furthermore, previous literature has emphasized a need for standardization in measurement and evaluation of engagement processes as methods to measure or evaluate citizen engagement are seldom utilized beyond the groups that develop them [42]. In light of our findings, we postulate this could occur due to (1) lack of accessibility (i.e., method difficult to find) or (2) lack of perceived applicability/modifiability (i.e., method viewed as unsuitable or too specific to a certain project or type of research and unmodifiable).

As per recommendations by Levac and colleagues, we invited citizen team members (BS, SL) to help interpret the findings of this scoping review and provide insights beyond those in the literature [27]. These citizen team members (BS, SL) remarked that empowerment, impact, respect, support, and value, common to frameworks identified by our study, were important to them in their experiences of participating in research. Additionally, they stated that the ability to openly communicate their concerns about the research project and their involvement has been important to them as members of a research team. Finally, they expressed a desire for an accessible lay resource to help people like them (i.e., citizens) be a meaningful part of research and stated that such a resource would vastly improve their comfort level with participating in research.

### Strengths and limitations

This scoping review was designed to form an evidence basis for future work to advance and standardize appraisal of citizen engagement in health research. This study has strengths and limitations to consider. Strengths of our scoping review include: (1) co-development of the study protocol with a multidisciplinary team including researchers, health professionals, and health sciences librarians, and (2) citizen involvement in its design and interpretation. These elements helped to create a comprehensive synthesis and discussion of the existing literature on measuring and evaluating citizen engagement in health research.

Our study also has limitations. A significant number of the methods we summarize in this scoping review are focus groups, interviews, and closed- and open-ended discussions and questions. These methods were often described in the literature with varying levels of detail, presenting difficulty in assessing the rigour of each method. While the level of detail available on included methods is variable, we do not perceive this as a limitation but rather an accurate snapshot of the currently utilized discussion-based methods to appraise citizen engagement in research. Another limitation to this study is possible unintended omittance of relevant literature due to (1) our definition of citizen engagement adapted from the CIHR [3], which may not align with all citizen engagement activities reported in the international literature, and (2) our approach to including only studies which discussed a method of measuring or evaluating citizen engagement as a major aim of the work. We recognize that as a scoping review designed to provide a high-level mapping of the literature, our search strategy will likely have missed some studies. Thirdly, we only searched and included peer-reviewed literature (i.e., omitted grey literature) around methods to measure and/or evaluate citizen engagement in health research to capture studies with higher methodological quality and minimize surplus complexity in the results. Lastly, like previous reviews of citizen engagement [74–77], much of the literature we captured reflects United Kingdom-based practices around citizen engagement in health research. This is due to targeted NIHR efforts to set standards for patient and public involvement [78] (i.e., citizen engagement), making the United Kingdom a leader in participatory health research. While this is a potential weakness to our study, we have clearly stated the geographical location of included studies to highlight any practices distinct to the United Kingdom, in order to avoid misrepresenting worldwide citizen engagement practices.

## Conclusions

While there has been an increase in published methods to measure and evaluate citizen engagement over the past decade, there remains a need for standardized guidelines on appraising citizen engagement in research. Extensive variation in terminology used around citizen engagement contributes to a lack of unified principles or criteria that comprise effective citizen engagement and development of a single set of core principles that indicate degree (i.e., measurement) and quality (i.e., evaluation) of citizen engagement is necessary. This set of principles could be impactful if further developed as guidelines to suit specific types of research (e.g., clinical, health services, pre-clinical) and varying audiences (i.e., citizens, patients, researchers, other stakeholders). Commitment to citizen engagement in research by funding bodies, research institutions, and scientific journals could create a shift in research culture promoting use of standardized practices, helping citizen engagement move away from tokenism into an efficient and unified process.

## Recommendations

- We recommend standardization of terminology (i.e., citizen engagement rather than a multitude of other terms) used to describe participation of lay individuals in health research.
- We recommend development of a specific framework for the measurement and evaluation of citizen engagement in health research, built to foster empowerment, impact, respect, support, and value in citizen engagement.

## Supplementary Information

**Additional file 1. Item S1**. Medline (Ovid) search strategy.

## Data Availability

Not applicable.
