# Supplementary material for: A scoping review of methods to measure and evaluate citizen engagement in health research
Source: Res Involv Engagem. 2022 Dec 10;8:72. doi: 10.1186/s40900-022-00405-2 (PMC9737710; doi:10.1186/s40900-022-00405-2)
Supplement: Supplementary file 1 — Additional file 1. Item S1. Medline (Ovid) search strategy. [file 40900_2022_405_MOESM1_ESM.docx]

**Item S1.** Medline (Ovid) search strategy.

**MEDLINE**

1. ((citizen* or consumer* or "lay person" or "lay people" or "lay community" or "lay group" or "lay member" or "community member*" or public or stakeholder*) adj3 (involv* or partner* or participation or participatory or collaborat* or contribution* or input or consultat* or engage* or empower* or advisor* or advising)).tw,kf.

2. Community Participation/

3. exp Community-Based Participatory Research/

4. co-design*.tw,kf.

5. 1 or 2 or 3 or 4

6. exp Biomedical Research/

7. exp Behavioral Research/

8. exp Public Health Systems Research/

9. exp Nursing Research/

10. exp Health Services Research/

11. ((biomedical or behavio?ral or clinical or medical or population health or public health or nursing or health) adj2 research).tw,kf.

12. 6 or 7 or 8 or 9 or 10 or 11

13. 5 and 12

14. Animals/ not humans/

15. 13 not 14

16. limit 15 to yr="2000 -Current"

**EMBASE**

1. ((citizen* or consumer* or "lay person" or "lay people" or "lay community" or "lay group" or "lay member" or "community member*" or public or stakeholder*) adj3 (involv* or partner* or participation or participatory or collaborat* or contribution* or input or consultat* or engage* or empower* or advisor* or advising)).ti,kw,ab.

2. community participation/ or citizen science/

3. co-design*.ti,kw,ab.

4. 1 or 2 or 3

5. exp medical research/

6. exp behavioral research/

7. exp public health systems research/

8. exp nursing research/

9. ((biomedical or behavio?ral or clinical or medical or population health or public health or nursing or health) adj2 research).ti,kw,ab.

10. 5 or 6 or 7 or 8 or 9

11. 4 and 10

12. Animals/ not humans/

13. 11 not 12

14. limit 13 to yr="2000 -Current"

**PSYCINFO**

1. ((citizen* or consumer* or "lay person" or "lay people" or "lay community" or "lay group" or "lay member" or "community member*" or public or stakeholder*) adj3 (involv* or partner* or participation or participatory or collaborat* or contribution* or input or consultat* or engage* or empower* or advisor* or advising)).mp.

2. community involvement/

3. co-design*.mp.

4. 1 or 2 or 3

5. exp public health research/

6. exp interdisciplinary research/

7. ((biomedical or behavio?ral or clinical or medical or population health or public health or nursing or health) adj2 research).mp.

8. 5 or 6 or 7

9. 4 and 8

10. Animals/ not humans/

11. 9 not 10

12. limit 11 to yr="2000 -Current"

**COCHRANE**

1. ((citizen* or consumer* or "lay person" or "lay people" or "lay community" or "lay group" or "lay member" or "community member*" or public or stakeholder*) adj3 (involv* or partner* or participation or participatory or collaborat* or contribution* or input or consultat* or engage* or empower* or advisor* or advising)).ti,kw,ab.

2. co-design*.ti,kw,ab.

3. 1 or 2

4. ((biomedical or behavio?ral or clinical or medical or population health or public health or nursing or health) adj2 research).ti,kw,ab.

5. 3 and 4

**CINAHL**

1. (MH "Citizen Science")
2. TI ( ((citizen* or consumer* or "lay person" or "lay people" or "lay community" or "lay group" or "lay member" or "community member*" or public or stakeholder*) N3 (involv* or partner* or participation or participatory or collaborat* or contribution* or input or consultat* or engage* or empower* or advisor* or advising)) ) OR AB ( ((citizen* or consumer* or "lay person" or "lay people" or "lay community" or "lay group" or "lay member" or "community member*" or public or stakeholder*) N3 (involv* or partner* or participation or participatory or collaborat* or contribution* or input or consultat* or engage* or empower* or advisor* or advising)) )
3. TI co-design* OR AB co-design*
4. S1 OR S2 OR S3
5. (MH "Research, Allied Health+") OR (MH "Behavioral Research") OR (MH "Clinical Research+") OR (MH "Embryo Research") OR (MH "Epidemiological Research+") OR (MH "Fetal Research") OR (MH "Genetic Research+") OR (MH "Health Services Research+")
6. TI ((biomedical or behavio?ral or clinical or medical or population health or public health or nursing or health) N3 research) OR AB ((biomedical or behavio?ral or clinical or medical or population health or public health or nursing or health) N3 research)
7. S5 OR S6
8. S4 AND S7
9. (MH "Animals")
10. (MH "Human")
11. S9 not S10
12. S8 not S11

**Web of Science**

1. (TS=(((citizen* or consumer* or "lay person" or "lay people" or "lay community" or "lay group" or "lay member" or "community member*" or public or stakeholder*) NEAR/3 (involv* or partner* or participation or participatory or collaborat* or contribution* or input or consultat* or engage* or empower* or advisor* or advising)))) OR TS=((co-design*))
2. ALL=(animal)
3. #1 NOT #2
4. TS=(biomedical or behavioral or behavioural or clinical or medical or nursing or public health or population health or health NEAR/2 research)
5. #3 AND #4
